# Dataset of clinical laboratory tests according to ordering variance among family physicians in Calgary, Alberta, Canada

**DOI:** 10.1016/j.dib.2019.104387

**Published:** 2019-08-12

**Authors:** Leonard T. Nguyen, Maggie Guo, Brenda Hemmelgarn, Hude Quan, Fiona Clement, Tolulope Sajobi, Roger Thomas, Tanvir C. Turin, Christopher Naugler

**Affiliations:** a Department of Pathology and Laboratory Medicine, Cumming School of Medicine, University of Calgary, Calgary, AB, Canada; b Department Community Health Sciences, Cumming School of Medicine, University of Calgary, Calgary, AB, Canada; c Department of Family Medicine, Cumming School of Medicine, University of Calgary, Calgary, AB, Canada

**Keywords:** Clinical laboratory tests, Utilization, Variance, Laboratory medicine, Family medicine

## Abstract

This data incorporates 2016 testing volumes ordered by family physicians and performed at Calgary Laboratory Services (CLS), the sole supplier of clinical laboratory services for the catchment area of the City of Calgary, Alberta, Canada. For each test, the mean number of tests ordered per patient was calculated over ordering Calgary physicians, along with arithmetic coefficients of variation (CV's). The latter parameter is reflective of variance in ordering practice among family physicians practicing in Calgary and is proposed as a benchmark measure for laboratory utilization in our accompanying research article [1]. The data table encompasses 358 tests ordered by at least 3 family physicians at a minimum total frequency of 100 within the 2016 study period and is presented in ascending order of rank in CV.

**Table:** Specifications Table

Subject area	*Biology*
More specific subject area	Laboratory Medicine, Healthcare Utilization
Type of data	Structured, tabular data
How data was acquired	Data extraction and analysis from clinical laboratory test volumes performed at CLS
Data format	Tabular with raw data and calculated values
Experimental factors	Data are presented for test volumes performed in 2016
Experimental features	Calculated parameters for each test include: 1) Median number of tests ordered per patient by each ordering physician 2) Average number of tests ordered per patient by each ordering physician 3) Arithmetic coefficient of variation, CV 4) 95% confidence interval lower and upper limits for CV
Data source location	Calgary Laboratory Services, Calgary, Alberta, Canada
Data accessibility	Data is included in this article
Related research article	L.T. Nguyen, M. Guo, B. Hemmelgarn, H. Quan, F. Clement, T. Sajobi, R. Thomas, T.C. Turin, C. Naugler, Evaluating practice variance among family physicians to identify targets for laboratory utilization management, Clin. Chim. Acta., 497, 2019, 1–5 [1].

**Table:** 

**Value of the Data** • The ranked list provides a full assessment of standardized ordering practice among family physicians in Calgary, a representative major North American city • Healthcare managers from other jurisdictions are encouraged to perform similar analyses and will find this data useful for comparative purposes • The calculation of CVs is easily adaptable to other fields and specialties to be applied as a benchmark parameter for utilization investigation

## 1 Data

From January 1 to December 31, 2016, over 10 million clinical tests were ordered by up to 1527 Calgary family physicians and performed at CLS. From these volumes, 358 hematology, microbiology or chemistry tests were ordered at least 100 times within the study period. For each of these tests, raw data columns are presented for total number of tests ordered (i.e. test volume, along with rank) and number of ordering physicians. Calculated fields include median and mean ordered tests between physicians, arithmetic coefficient of variation (CV, along with rank) and 95% confidence interval limits for the CV. Truncated selections of this table are presented in the accompanying research article [1].

## 2 Experimental design, materials, and methods

Test volumes within the study period of January to December 2016 by family physicians for community-based patients were extracted from the Laboratory Information System at CLS and summed up as total tests ordered and number of ordering physicians. For each test, the median and mean number of tests ordered per patient per ordering physician were determined. This required the number of tests ordered by each physician and their patient load, both of which are not disclosed here in order to protect data privacy. From the aggregated parameters for each test, the arithmetic CV and 95% confidence interval limits were calculated and the tests were ordered according to CV. For further detail on calculations, please refer to Ref. [1]. Tests that were ordered less than 100 times and/or by less than 3 physicians were excluded. Clinical laboratory tests included hematology, chemistry and microbiology tests.
